# [Retracted] LINC01094 promotes the invasion of ovarian cancer cells and regulates the Wnt/β‑catenin signaling pathway by targeting miR‑532‑3p

**DOI:** 10.3892/etm.2024.12545

**Published:** 2024-04-22

**Authors:** Haiyan Chen, Yanlin Liu, Ping Liu, Qiuxiang Dai, Peiliang Wang

Exp Ther Med 22:1228, 2021; DOI: 10.3892/etm.2021.10662

Following the publication of this paper, it was drawn to the Editor’s attention by a concerned reader that certain of the Transwell invasion assay data shown in Fig. 4G and wound-healing assay data in Fig. 4E on p. 8 were strikingly similar to data appearing in different form in other articles written by different authors at different research institutes, which had either already been published or were under consideration for publication at around the same time. Owing to the fact that the contentious data in the above article had already been published prior to its submission to *Experimental and Therapeutic Medicine,* the Editor has decided that this paper should be retracted from the Journal. The authors were asked for an explanation to account for these concerns, but the Editorial Office did not receive a satisfactory reply. The Editor apologizes to the readership for any inconvenience caused.

